# Presentation of a new mutation in FMF and evaluating the frequency of distribution of the MEFV gene mutation in our region with clinical findings

**DOI:** 10.1007/s11033-020-06040-y

**Published:** 2021-03-18

**Authors:** Abdullah Arpacı, Serdar Doğan, Hazal Fatma Erdoğan, Çiğdem El, Sibel Elmacıoğlu Cura

**Affiliations:** 1 Department of Medical Genetics, Hatay Mustafa Kemal University Faculty of Medicine, Alahan, Tayfur Sökmen Campus, 31001 Antakya, Hatay Turkey; 2 Department of Medical Biochemistry, Hatay Mustafa Kemal University Faculty of Medicine, Antakya, Hatay Turkey; 3 Department of Child Health and Diseases, Hatay Mustafa Kemal University Faculty of Medicine, Antakya, Hatay Turkey

**Keywords:** FMF, R202Q, Tel Hashomer criteria

## Abstract

Familial Mediterranean Fever (FMF), which is an autosomal recessive disease characterized by recurrent self-limiting fever, peritonitis, pleuritis, arthritis and erysipelas-like erythemas, has been common among ethnic groups such as Turkish, Armenian, Arabic and Jewish. The clinical presentation is caused by mutations in the MEFV gene encoding the Pyrin protein. In this study, we aimed to present a new mutation that has not been previously defined from the mutations in the MEFV gene which is responsible for the genetic pathology of familial Mediterranean fever and to evaluate the frequency of distribution of the MEFV gene mutation among different ethnic groups living in our region. In present retrospective study, a total of 2639 clinically suspected FMF patients who were referred to Hatay Mustafa Kemal University Hospital between 2010 and 2017 were recorded. MEFV gene mutations were observed using DNA sequence analysis. MEFV mutations were found in 2079 of the 2639 patients (78.7%) Among these patients 184 (6.97%) were homozygous, while 1365 (51.72%) were heterozygous. The most frequently observed mutation was R202Q (1319, 19.55%) followed by E148Q (n = 476, 7.05%), M694V (n = 439, 6.51%), V726A (n = 146, 2.16%) and M680I (n = 135, 2%). In a case clinically diagnosed as FMF, a new mutation called S145G (p. Ser145Gly, c.433A > G) was identified in exon 2 of the MEFV gene. Besides, addition of a new pathogenic MEFV variant to the literature, the relationship between the FMF clinic and homozygous form of R202Q, which was previously considered as a polymorphism, was highlighted.

## Introduction

Familial Mediterranean Fever (OMIM: 249100,FMF) is an autosomal recessive disease characterized by recurrent self-limiting fever, peritonitis, pleuritis, arthritis and erysipelas-like erythemas [1]. It has been common among ethnic groups such as Turkish, Armenian, Arabic and Jewish. FMF prevalence in Turkey is about 1:400 to 1:1000. It is estimated that Turkey has more than 100,000 patients with FMF and the carrier rate is 1:5 [2–4]. Tel Hashomer criteria and Simplified FMF diagnosis criteria suggested by Livneh et al. are used in clinical diagnosis [5, 6]. Acute phase reactants such as erythrocyte sedimentation rate (ESR), C-reactive protein (CRP), fibrinogen, haptoglobin, C3, C4 and clinical symptoms are supported by ethnic origin and family history. However, many patients presenting with atypical attacks may be difficult to diagnose and may cause delay in treatment. MEFV (Mediterranean fever) genetic testing is used as a diagnostic aid, especially in atypical cases [7]. The most serious complication of FMF is the development of serum amyloid A (SAA) amyloidosis, which primarily affects the kidneys but may also include other organs. It is more prevalent in Armenians (28.1%), Jews of Israel (24.2%), Turks (21.5%) and less in Arabs, and Iranian Azeri Turks, Syrians. Renal amyloidosis accounted for 35% and 60% of deaths in men and women in a study conducted in Israel. The preferred treatment for renal amyloidosis is colchicine therapy. The reason for the low prevalence of FMF-related amyloidosis in Arabs may be the collection of these data after colchicine was used as standard therapy [8–12].

The MEFV gene encodes a protein of 781 amino acids named Pyrin/Marenostrin. The pyrin gene is mostly expressed in monocytes, neutrophils, eosinophils and fibroblasts. The function of pyrin is to suppress inflammation by autophagy of innate immune regulators. The mutation product, pyrin variants in FMF patients, progress through neutrophil activation and uncontrolled interleukin-1 (IL-1) production and causes inflammation that self-limiting and repetitive in all serous membranes, especially peritoneum, pleura and joints [8]. Differences in the clinical cases and development of amyloidosis are affected by the type of MEFV mutations. The gene that causes FMF (Mediterranean fever gene, MEFV) is found in the short arm of chromosome 16p13.3 and consists of 10 exons separated by 9 introns. Mutations have been found especially in exons 2, 3, 5 and 10 of the MEFV gene. The MEFV online database infevers shows more than 377 alleles defined as mutations and polymorphisms [13]. The most common mutations are M694V, M680I, V726A, M694I in exon 10 and E148Q, E148V in exon 2. M694V homozygous mutation is with more severe clinic feature than other mutations. Five founder mutations, V726A, M694V, M694I, M680I and E148Q account for 74% of FMF genotypes from typical cases (Armenians, Arabs, Jews, and Turks) [14].

In the Mediterranean region, which includes our province, FMF is very common and the frequency of mutations varies according to each region. All genetic mutations that cause clinical FMF have not been clarified. Therefore, in present study our aim is to investigate the frequency and clinical characteristics of patients in our province and to find new mutations that may cause disease in patients diagnosed with clinical FMF.

## Material and method

A total of 2639 clinically suspected FMF patients referred to Hatay Mustafa Kemal University Hospital between 2010 and 2017 were enrolled in this study. The demographic data of the patients were collected through the Hospital Information Management System (HIS).

Genomic DNA was isolated from the whole blood sample with EDTA using the isolation kit (Macherey-Nagel GmbH & amp; Co.KG, Germany). In PCR amplification, forward and reverse primers were used in 4 different tubes for Exon 2, Exon 3, Exon 5 and Exon 10 MEFV gene regions. Amplification samples were sequenced according to the ABI PRISM BigDye Terminator Cycle Sequensing Ready Reaction Kit (Applied Biosystems, Foster City, CA, USA). Sequence reactions were analyzed by automatic fluorescence radiated sequence reader (ABI PRISM 3500, Applied Biosystems). Mutations were confirmed by the sequence appearance of antisense DNA strands.).

All statistical calculations were performed using the SPSS 22 (IBM SPSS Statistics for Windows, Version 20.0. Armonk, NY: IBM Corp). Statistical significance for all analyses was accepted of p < 0.05. Categorical values were expressed as percentages, frequency and non-categorical variables were given as the mean ± standard deviation (SD). Chi-square tests were used to analyze categorical data. Student t test and Mean Whitney U test were used to analyze continuous data.

Informed consent to participate was not obtained from the subjects as it was a retrospective study based on archive scanning. Permission was obtained from the Chief Physician of the Hatay Mustafa Kemal University (HMKU) Health Practice and Research Hospital for the use of genetic data results and clinical features. In addition, the study was approved by the Ethics Committee of the HMKU Faculty of Medicine (2018/138). We recalled the patient with the new mutation and confirmed the patient's mutation and clinical findings. Informed consent was obtained from this patient.

## Results

A total of 2639 patients (1374 female, 1265 male, aged between 0 and 82) enrolled in this study. The mean age of patients was 16.5 ± 14.2 years. Out of 2639 patients, 1793 (67.9%) were in pediatric group (< 18 years) and 846 (32.1%) were in adult (≥ 18 years) group. The rate of alleles carrying one of the identified mutations was 39.7% in 6748 total alleles coming from 2639 suspected FMF patients; and 45 different genotypes and 24 different mutations, including a new mutation, were detected in these patients. The MEFV mutations in patients are shown in Table 1. MEFV mutations were found in 2079 of the 2639 patients (78.7%). Among these patients 184 (6.97%) were homozygous, while 1365 (51.72%) were heterozygous. The compound heterozygous mutation was detected in 340 (12.88%) patients while complex alleles were 188 (7.12%). The MEFV mutation frequency was presented in Table 2. The most frequently observed mutation was R202Q (1319, 19.55%) followed by E148Q (n = 476, 7.05%), M694V (n = 439, 6.51%), V726A (n = 146, 2.16%) and M680I (n = 135, 2%). E148 homozygote mutation was found to be significantly higher in children than in adults (p < 0.05). In other mutations, significance was not found between age and frequency of mutation. The most common clinical symptoms of the patients were abdominal pain (97.89%) and fever (92.46%).

**Table 1:** The MEFV mutation genotype distribution of patients

Mutation	n	%
Homozygous		
R202Q homozygous	107	4.05
E148Q homozygous	27	1.02
M680I homozygous	19	0.72
M694V homozygous	14	0.53
M694I homozygous	11	0.42
V726A homozygous	3	0.11
G196W homozygous	2	0.08
A744S homozygous	1	0.04
Total	184	6.97
Heterozygous		
R202Q heterozygous	795	30.13
E148Q heterozygous	318	12.05
M694V heterozygous	81	3.07
V726A heterozygous	53	2.01
M680I heterozygous	35	1.33
A744S heterozygous	26	0.99
M694I heterozygous	12	0.45
R761H heterozygous	12	0.45
K695R heterozygous	11	0.42
R241K heterozygous	6	0.23
E167D heterozygous	3	0.11
M680L heterozygous	3	0.11
D661N heterozygous	2	0.08
G632A heterozygous	2	0.08
A744T heterozygous	1	0.04
E125E heterozygous	1	0.04
N766H heterozygous	1	0.04
T681I heterozygous	1	0.04
V659F heterozygous	1	0.04
L110P heterozygous	1	0.04
Total	1365	51.72
Compound heterozygous		
R202Q heterozygous/M694V heterozygous	126	4.77
R202Q heterozygous/E148Q heterozygous	59	2.24
V726A heterozygous/M680I heterozygous	27	1.02
E148Q heterozygous/M694V heterozygous	18	0.68
R202Q heterozygous/M680I heterozygous	17	0.64
R202Q heterozygous/A744S heterozygous	14	0.53
R202Q heterozygous/V726A heterozygous	12	0.45
M694V heterozygous/V726A heterozygous	11	0.42
M694V heterozygous/M680I heterozygous	10	0.38
E148Q heterozygous/R761H heterozygous	6	0.23
E148Q heterozygous/V726A heterozygous	6	0.23
M694I heterozygous/V726A heterozygous	4	0.15
E148Q heterozygous/P706P heterozygous	3	0.11
M680I heterozygous/A744S heterozygous	3	0.11
R202Q heterozygous/R761H heterozygous	3	0.11
E148Q heterozygous/A744S heterozygous	2	0.08
E148Q heterozygous/M694I heterozygous	2	0.08
M694V heterozygous/R761H heterozygous	2	0.08
R202Q heterozygous/G632A heterozygous	2	0.08
A744S heterozygous/R761H heterozygous	1	0.04
E148Q heterozygous/G632A heterozygous	1	0.04
E148Q heterozygous/K695R heterozygous	1	0.04
E148Q heterozygous/M680I heterozygous	1	0.04
E148Q heterozygous/R241K heterozygous	1	0.04
M680I heterozygous/R761H heterozygous	1	0.04
M694V heterozygous/A744S heterozygous	1	0.04
M694V heterozygous/M694I heterozygous	1	0.04
R202Q heterozygous/K695R heterozygous	1	0.04
R202Q heterozygous/M694I heterozygous	1	0.04
R202Q heterozygous/ V704I heterozygous	1	0.04
V726A heterozygous/R761H heterozygous	1	0.04
E148Q heterozygous/L110P heterozygous	1	0.04
Total	340	12.88
Complex allels		
R202Q homozygous/M694V homozygous	61	2.31
R202Q heterozygous/M694V heterozygous/V726A heterozygous	22	0.83
R202Q homozygous/M694V heterozygous	22	0.83
R202Q heterozygous/E148Q heterozygous/M694V heterozygous	17	0.64
R202Q heterozygous/M694V heterozygous/M680I heterozygous	16	0.61
R202Q heterozygous/M694V homozygous	15	0.57
R202Q heterozygous/E148Q heterozygous/M694V heterozygous	5	0.19
R202Q heterozygous/M694V heterozygous/R761H heterozygous	5	0.19
R202Q heterozygous/V726A heterozygous/M680I heterozygous	4	0.15
R202Q heterozygous/E148Q heterozygous/M694V heterozygous	2	0.08
R202Q heterozygous/ M694V heterozygous/M694I heterozygous	2	0.08
E148Q heterozygous/V726A heterozygous/M680I heterozygous	1	0.04
E148Q heterozygous/V726A heterozygous/R761H heterozygous	1	0.04
E148Q homozygous/M694V heterozygous	1	0.04
G196W heterozygous/M694I homozygous	1	0.04
M694V homozygous/R761H heterozygous	1	0.04
R202Q heterozygous/E148Q heterozygous/M694V homozygous	1	0.04
R202Q heterozygous/E148Q heterozygous/E230K heterozygous	1	0.04
R202Q heterozygous/E148Q homozygous	1	0.04
R202Q heterozygous/M694V heterozygous/A744S heterozygous	1	0.04
R202Q heterozygous/M694V heterozygous/ M680I heterozygous	1	0.04
R202Q heterozygous/R241K heterozygous/M694V heterozygous	1	0.04
nnR202Q heterozygous /M694V homozygous	1	0.04
R202Q homozygous/M694V heterozygous	1	0.04
R202Q homozygous/E148Q heterozygous	1	0.04
R202Q homozygous/V726A heterozygous	1	0.04
R241K heterozygous/M694I homozygous	1	0.04
Total	188	7.12
Number of patients with identified mutations	2076	78.7
Number of patients with no identified mutations	563	21.3
Total patient number	2639	100.0

**Table 2:** The MEFV mutation frequencies in present study

Mutation type	n	% (total mutation)	% (total allel)
Common mutations			
R202Q	1319	49.29	19.55
E148Q	476	17.79	7.05
M694V	439	16.41	6.51
V726A	146	5.46	2.16
M680I	135	5.04	2.00
A744S	49	1.83	0.73
R761H	33	1.23	0.49
M694I	35	1.31	0.52
Rare mutations			
K695R	12	0.45	0.18
R241K	9	0.34	0.13
E167D	3	0.11	0.04
M680L	3	0.11	0.04
S179N	3	0.11	0.04
G196W	3	0.11	0.04
D661N	2	0.07	0.03
L110P	2	0.07	0.03
A744T	1	0.04	0.01
E125E	1	0.04	0.01
G632A	1	0.04	0.01
N766H	1	0.04	0.01
T681I	1	0.04	0.01
V659F	1	0.04	0.01
V704I	1	0.04	0.01
Total	2676	100.00	39.66

Arthritis, arthralgia, and myalgia were more common in patients with R202Q mutations. While the frequency of abdominal pain was similar among mutations, the rate of appendectomy was more common in patients with R202Q mutations. Erysipelas-like erythema were more common in patients with E148Q mutations. Vasculitis was similar in frequency among the mutations. All clinical findings of the patients are summarized in Table 3 together with their genotypes.

**Table 3:** Comparison of the clinical features of the patients with their genotypes

Clinical symptoms	R202Q n (%)	E148Q n (%)	M694V n (%)
Abdominal pain	877 (97.22)	302 (87.53)	93 (97.89)
Fever	743 (82.37)	319 (92.46)	84 (88.42)
Arthralgia	569 (63.08)	208 (60.28)	47 (49.47)
Myalgia	692 (76.71)	228 (66.08)	56 (58.94)
Headache	317 (35.14)	97 (28.11)	29 (30.52)
Arthritis	278 (30.82)	71 (20.57)	21 (22.10)
ELE^a^	241 (26.71)	28 (81.15)	14 (14.73)
Chest pain	124 (13.74)	17 (4.92)	20 (21.05)
Renal failure^b^	43 (47.67)	2 (0.57)	6 (6.31)
Appendectomi	98 (10.86)	2 (0.57)	5 (5.26)
Vasculitis^c^	94 (10.42)	37 (10.72)	12 (12.63)

^a^Erysipel-like erythema

^b^Amyloidosis

^c^HSP

## Discussion

MEFV mutation predominantly affects people living in or originating from areas around the Mediterranean basin, mainly Arabs, Armenians, Turks and Jews. The most common mutations in Arabs are V726A, M680I and M694V, while in Armenians this order is seen as M694V, M680I, and V726 [4, 14]. In a large study of 1387 patients in Egypt, the most common mutations were E148Q (38.6%), M694I (18.1%) and V726A (15.8%) [15]. In the Jewish population, different mutation distributions are observed according to different ethnic origin. M694V and E148Q in the Jews of North Africa, E148Q, V726A in the Askenazi Jews are more common. In a study conducted in Azeri Turks in Iran, M694V (40.2%), E148Q (13.7%) and V726A (13.7%) mutations were observed [16]. Although Mutation Frequency shows more variability according to regions in Turkey, the most common mutations are M694V M680I, V726A, E148Q [2–4]. The reason of the differences in mutation carriage rate might arise from geographical differences among the patients from the all studies of Turkey. In addition, MEFV gene frequency were evaluated for suspected patients or clinical diagnosed patients (Tel Hashomer, Simplified FMF diagnosis criteria) in various studies from Turkey. Therefore, it is assumed that the differences in MEFV gene mutation frequency are caused by these conditions. In our study of 2639 clinically suspected FMF patients the most common MEFV mutations were R202Q (19.55%), E148Q (7.05%), M694V (6.51%), V726A (2.16%), and M680I (2%) (Table 2). Our patients with R202Q mutation had clinical findings and benefited from colchicine treatment hence we accepted R202Q as a mutation similarly studies by Barut et al., Comak et al., Gumus, Kılınc et al. (Table 3) [17–20]. However, in some other articles, R202Q is still not considered as a mutation [21–24].

The most common mutation was R202Q which was detected in 1319 patients and observed in 19.55% of alles in our study while it was found to be 59.6%, 23.7%, 21.4% in studies by Yigit et al., Kocakap et al., Günesacar et al. [25–27]. The frequencies of homozygous, heterozygous, compound heterozygous and complex alleles of the R202Q mutation were 4.05% (n = 107), 30.13% (n = 795), 8.94% (n = 236) and 6.86% (n = 181), respectively in present study. Out of the distribution of patients, the most common cases consisted of patients with heterozygous R202Q (30.13%) and heterozygous E148Q (12.05%) (Table 1). The most frequently observed compound heterozygote and complex alleles were R202Q heterozygote/M694V heterozygote (n = 126, 6.01%) and R202Q Homozygote/M694V Homozygous (n = 61, 2.31%).

R202Q mutation was reported as a prevalent polymorphism. However, in recent studies, a higher frequency of R202Q was found among patients with FMF compared to healthy controls, suggesting that R202Q may be a disease-causing mutation [28–30]. In the study of Ritis, while homozygous R202Q was detected in four of 26 FMF patients, no R202Q homozygous mutation was found in any of 60 healthy individuals and the difference between groups was statistically significant [31]. Similarly, while R202Q heterozygote frequency was similar between patient and healthy groups in a study by Yigit et al., homozygous R202Q was significantly higher in FMF patients compared to healthy group. Comak et al. found that seven patients (23.3%) with the R202Q mutation had typical FMF episodes. Two of these FMF patients (3.6%) had heterozygous R202Q mutation. In 19 patients (63.3%) with homozygous R202Q mutation, at least one symptom of abdominal pain, fever, arthralgia/myalgia, arthritis or chest pain was observed [19]. In addition, Ozturk et al. presented R202Q homozygote mutation in two amyloidosis patients and R202Q homozygote mutation was not found in the healthy group [26, 30, 32]. Thus, the homozygote form of R202Q may be a risk factor in the development of FMF clinic. Furthermore R202Q (c.605G > A) was reported to be in linkage disequilibrium with M694V [13]. The most frequent compound heterozygous was R202Q/M694V genotype in the present study. But FMF patients have a higher frequency R202Q mutation than M694V frequency, therefore there might be FMF patients carrying R202Q without linkage disequilibrium with M694V [30]. In our study, it was detected that the most common mutation in our region was R202Q, the clinical findings of these patients were similar to the diagnostic clinical findings of FMF reported in the literature, and all patients responded to colchicine treatment [33, 34]. In this study, unnecessary surgical intervention (appendectomy) and renal failure were found to be more common in patients with homozygous and heterozygous R202Q mutations. In addition, other clinical findings of FMF patients with R202Q mutation were seen with a similar frequency to patients with the M694V mutation (Table 3). These data support that R202Q may be mutation rather than polymorphism. Thus, we think that the R202Q may be a risk factor in the development of the FMF clinic and R202Q mutation analysis should be added to the routine molecular diagnosis of FMF patients.

*Remarkably*, in this study unlike other studies; we detected the S145G mutation which it has the feature of not being defined previously in a case that clinically diagnosed as FMF and benefiting from colchicine treatment. It was learned that the case has frequency of attacks of 4–8 weeks, seen as fever and abdominal pain also received response from colchicine treatment as in patients with other defined mutations of the gene. In the case clinically diagnosed as FMF, but who has without frequent mutations of the MEFV gene, the whole exome sequence analysis was performed and a new mutation called S145G (p.Ser145Gly, c.433A>G) was identified in exon 2 of the MEFV gene (https://infevers.umai-montpellier.fr/web/detail_mutation.php). There is no biochemical test that can make the definitive diagnosis of FMF and the diagnosis is based on clinical findings (Tel Hashomer criteria). Therefore, it causes many examinations, which worrying, tiring the patients and their families and costly. Further it may cause unnecessary surgical interventions in some of these patients. Generally, the screening of the most common mutations in order to diagnose confirms the FMF diagnosis of the majority of cases in clinical practice. So, determining regional mutations is very important in order not to misdiagnose patients. However, for confirm the diagnosis in the rarely cases which they have atypical clinical finding and the most common mutations are not available, it is considered that 'whole sequence analysis' is recommended [35, 36].

The second most common mutation was E148Q which was described as a disease causing mutation with low penetrance and mild symptoms in literature [4, 21, 37]. In our study, the frequency of unnecessary surgical intervention due to severe abdominal pain and renal failure was found moderate low in patients with E148Q mutation. This result may be related to the milder FMF clinic in patients with E148Q mutation. However, skin findings in the form of erysipel-like erythema were more common in these patients. Therefore, we think that FMF should be kept in mind in atypical cases presenting with recurrent erythematous skin findings and especially without severe abdominal pain (Table 3). E148Q was the most second frequent mutation between 8.9% to 26.88% reported similarly in other studies by Kilinc et al., Oztuzcu et al., Cekin et al. [20–22] while it was reported the most common mutations with 34.1%, 30.8% and 30.7% in studies by Yeşilada et al., Evliyaoğlu et al. and Ece et al. [37–39]. M694V is the third most common mutation (6.51%) in this study, similar to Kılınç et al. and Güneşacar et al. [20, 27]. The studies made by Evliyaoglu et al., Yesilada et al., Coskun et al. and Gumus reported M694V as the second most common mutation [18, 38–40]. M694V was the most commonly observed mutation, which was found between 14.7% to 53.8% of the MEFVs alleles in Turkish Patients with FMF [2, 3, 17, 22–24, 40–48]. In our study, the third and fourth most commonly seen mutations were V726A and M680I G/C which were observed 2.16% and 2% of carrier alleles, respectively. The rare mutations are listed in Table 2. During molecular analysis of MEFV gene in our laboratory, P708T, G296A, H739N, I247L, I640F, M470V variants were detected in the literature for the first time in FMF patient.

We detected at least one mutation in 2079 (78.77%) of the 2639 patients in our study group. The ratio of finding mutation in patients ranged between 45.6 and 67.7% in other studies from Turkey [3, 20, 21, 24, 25, 44, 45]. When the patients are evaluated in terms of MEFV gene mutation genotypes, it is remarkable that the most common mutation in both the homozygous and heterozygous genotype group was R202Q. In addition, when the clinical findings of the patients were compared with their genotype groups, the fact that the findings were found at similar rates in M694V mutation in both homozygous and heterozygous groups, it supports our opinion that R202Q should be considered as one of the responsible mutations from FMF (Table 3). This result may have been higher than other studies because we considered R202Q as a mutation in our study. Another studies may be that these patients are early diagnosed because of that with the frequent occurrence of the disease in Hatay. The limitation of our study is the absence of a healthy patient group. Although all patients have typical FMF clinical findings, studies comparing R202Q ratio with healthy group are needed.

In conclusion, we think that the R202Q mutation analysis should be added to the routine molecular diagnosis of FMF patients, and that the screening of the new mutation (S145G) we presented before the whole sequence analysis in the evaluation of selected cases will be both more economical and faster. Additionally, the three most common mutations in this study were R202Q (19.55%), E148Q (7.05%), and M694V (6.51%), respectively. The distribution rate of MEFV mutations differs from other regions where FMF is prevalent in the world, because the study group consists of many ethnic groups living in our region and confirms the mutational heterogeneity of FMF. Because of the risk of complications such as amyloidosis in patients with FMF, physicians should not waste time diagnosing the disease and initiating treatment.

## Data Availability

The datasets used for the current study are available from the corresponding author on reasonable request.

## Abbreviations

FMF: Familial Mediterranean fever
MEFV: Mediterranean fever
ESR: Erythrocyte sedimentation rate
CRP: C-reactive protein
SAA: Serum amyloid A
HIS: Hospital Information Management System
